# Fano Resonance Based on Metal-Insulator-Metal Waveguide-Coupled Double Rectangular Cavities for Plasmonic Nanosensors

**DOI:** 10.3390/s16050642

**Published:** 2016-05-05

**Authors:** Zhidong Zhang, Liang Luo, Chenyang Xue, Wendong Zhang, Shubin Yan

**Affiliations:** 1 Science and Technology on Electronic Test & Measurement Laboratory, North University of China, No. 3 Xueyuan Road, Taiyuan 030051, China; zdzhang@nuc.edu.cn (Z.Z.); 15135165573@163.com (L.L.); xuechenyang@nuc.edu.cn (C.X.); wdzhang@nuc.edu.cn (W.Z.); 2 Key Laboratory of Instrumentation Science & Dynamic Measurement, Ministry of Eduction, North University of China, No. 3 Xueyuan Road, Taiyuan 030051, China

**Keywords:** surface plasmon polaritons, metal-insulator-metal waveguide, Fano resonance, refractive index sensor, finite element method

## Abstract

A refractive index sensor based on metal-insulator-metal (MIM) waveguides coupled double rectangular cavities is proposed and investigated numerically using the finite element method (FEM). The transmission properties and refractive index sensitivity of various configurations of the sensor are systematically investigated. An asymmetric Fano resonance lineshape is observed in the transmission spectra of the sensor, which is induced by the interference between a broad resonance mode in one rectangular and a narrow one in the other. The effect of various structural parameters on the Fano resonance and the refractive index sensitivity of the system based on Fano resonance is investigated. The proposed plasmonic refractive index sensor shows a maximum sensitivity of 596 nm/RIU.

## 1. Introduction

In the direction perpendicular to the metal-insulator interface, the surface plasmon polaritons (SPPs) energy shows an exponential decay function [1,2]. As a result, the SPPs are strongly confined to near the metal-insulator interface, and can overcome the traditional optical diffraction limitation [3,4,5]. The strong SPPs at metal-dielectric interfaces can enhance molecular signals [6,7], making it useful in high-sensitivity sensors such as temperature sensors [8], biosensors and chemical sensors [9,10,11]. In addition, SPPs are regarded as a promising information carrier for next-generation ultrahigh-density photonic integrated circuits [12].

Metal-insulator-metal (MIM) waveguide-coupled resonators have attracted considerable research interest because of their easy on-chip integration and deep-subwavelength confinement of light [13,14]. Many photonic devices based on MIM waveguides have been designed and investigated, such as filters [15], wavelength division multiplexers [16], and high-sensitivity plasmonic sensors [17]. Plasmonic sensors based on MIM waveguides [13,14,18] have received considerable research interest because of the need for ultrahigh-sensitivity biochemical sensors [19,20]. Plasmonic devices based on Fano resonance exhibit great sensitivity and large figure of merit (FOM) [14,21]; these properties make Fano resonance appealing for use in sensors, lasing, switching, nonlinear and slow-light devices [18,22,23,24]. The plasmonic refractive index sensor with diverse structure are investigated, such as MIM waveguide coupled only resonator [25,26,27] and MIM waveguide coupled composite resonator [28,29]. Although the plasmonic sensor own the advantage of small size, and ease to integration, but its sensitivity is not as high as that of fiber sensor (30,100 nm/RIU) [25]. Therefore, how to optimize the plasmonic refractive index structure design to improve the sensitivity is a key issue for designing plasmonic sensor. Fano resonance is extremely sensitive to changes in the refractive index because it is a weak interference phenomenon that has unique line shape [30,31], which provides a very promising pathway to achieve ultrahigh sensitivity sensor. Fano resonances can be obtained from the interaction between the narrow discrete modes and broad continuous modes inside a subwavelength cavity [24].

In this paper, we design a plasmonic refractive index nanosensor based on a MIM waveguide-coupled double rectangular cavities, which is composed of two MIM waveguides and double rectangular cavities. Compared with other sensors, SPPs sensors have an inherent advantage to achieve high integration. The transmission spectrum and magnetic field distributions of the nanosensor are simulated using the finite element method (FEM) with a perfectly matched layer (PML) absorbing boundary condition. The effects of the structural parameters of the MIM waveguide-coupled double rectangular cavity on the propagation properties of the nanosensor are studied. The proposed structure can be easily integrated with various photonic devices and chips.

## 2. Structural and Analytical Method

**Figure 1:**
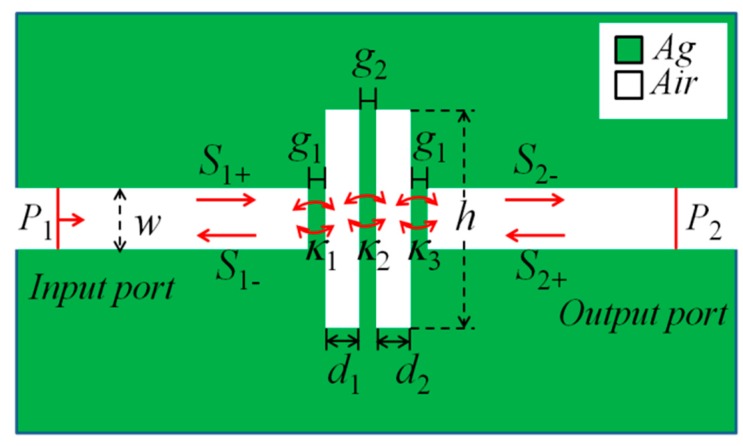
Schematics of the proposed MIM waveguide-coupled double rectangular cavities.

Considering that three-dimensional (3D) model simulation requires too many computer resources and the limitations of our computer workstation, the two-dimensional (2D) plasmonic waveguide coupled resonator system was investigated by the FEM in this paper. The working principle of the 2D model as similar to that of the real 3D model, because in real photonic devices if the depth of the metal layer is large enough *(i.e*., the third dimension much larger than the light wavelength), then the properties of the 3D model can be approximated by a 2D model. Figure 1 illustrates the geometry of the designed 2D MIM waveguide-coupled double rectangular cavity, which consists of two straight MIM waveguides with one end sealed and double rectangular cavities with a parallel arrangement. One rectangular cavity behaves as the active cavity, and the other rectangular cavity as the passive cavity. To ensure fundamental transverse magnetic (TM_0_) mode propagation in the MIM waveguide, the insulator layer width of the MIM waveguide was fixed at *w* = 50 nm [32]. The input port (*P*_1_) and output port (*P*_2_) are located in the left and the right end of the MIM waveguide, respectively. The coupling distance between the MIM waveguide and rectangular cavity is *g*_1_ (5 nm) and the gap between the double rectangular cavities is *g*_2_. The widths of the left and right rectangular cavities are *d*_1_ and *d*_2_, respectively. The height of the double rectangular cavities is *h*. The refractive index of the dielectric layer (white) in the MIM waveguide and rectangular cavity is denoted as *n*.

The propagation properties of the MIM waveguide-coupled double rectangular cavities were simulated by the FEM. PMLs were set at the up and bottom boundaries of the structure. The frequency-dependent complex relative permittivity ε(ω) of silver is characterized by the modified Debye-Drude dispersion model [33]:

(1) $$\varepsilon \left(\omega \right)=\varepsilon_{\infty }+\left(\varepsilon_{s}-\varepsilon_{\infty }\right)/\left(1+i\omega \tau \right)+\sigma /i\omega \varepsilon_{0}$$

where the infinite permittivity ε_∞_ = 3.8344, static permittivity ε_s_ = −9530.5, relaxation time τ = 7.35 × 10^−15^, and conductivity σ =1.1486 × 10^7^ s/m.

For MIM waveguide coupling resonator, the resonance wavelength can be determined by [34,35]:

(2) λm=2Re(neff)Lm−ψr/π(m=1,2,⋯)

where *L* is the perimeter of the cavities, positive integer *m* is the number of antinodes of the standing SPP wave, and ψ_r_ is the phase shift of the beam reflected at one end of the cavity. Re(*n*_eff_) is the real part of the effective refractive index in MIM waveguide, which can be obtained by solving the dispersion relation of the TM_0_ mode in a MIM waveguide.

The TM_0_ model equation in the MIM waveguide is [15,32]:

(3) $$tanh\;\left(kd\right)=-2kp\alpha_{c}/\left(k^{2}+p^{2}{\alpha_{c}}^{2}\right)$$

where *k* and *d* are the wave vector in the waveguide and the width of the MIM waveguide, respectively. The parameters *p* and *α*_c_ in Equation (3) are defined as *p* = ε_in_/ε_m_ and α_c_ = [*k*_0_^2^ (ε_in_ − ε_m_) + κ]^1/2^. ε_in_ and ε_m_ are the dielectric constants of the insulator and metal, respectively. The wave vector in free space *k*_0_ = 2π/λ_0_. Therefore, *k* can be solved from Equation (3) using the iterative method. Thus, the effective index neff of the MIM waveguide can be expressed as Re(*n_eff_*) = [ε_m_ + (*k*/*k*_0_)^2^]^1/2^. The wavelength of SPPs λ_spp_ can be obtained from λ_spp_ = λ_0_/Re(*n_eff_*). *P*_1_ is defined as the input port, and *P*_2_ is the output port. The transmission of the MIM waveguide coupling cavity is determined as *T* = (*S*_21_)^2^, where *S*_21_ is the transmission coefficient from port 1 to port 2. In addition, FOM was used to evaluate the sensitivity of the refractive index sensor. FOM is defined as (δλ/δ*n*)/FWHM, where δλ is the wavelength change corresponding to the refractive index change δ*n*, and FWHM is the full width at half-maximum of the resonance peak.

For the MIM waveguide coupled double rectangular cavities system, the temporal coupled mode theory (CMT) [36,37] is utilized to analyze in detail the Fano resonance in this paper. The amplitudes of the SPPs wave of the cavity are denoted by *S_i_* ± (*i* = 1, 2, and 3), and the subscripts ± of S*_i_*± denote the input and output from the rectangular cavity (as shown in Figure 1), respectively. When an optical wave with a frequency ω is launched only from the input port of the MIM waveguide (S_2+_ = 0), the time evolution amplitude *A_i_* ± (*i* = 1,2, and 3) of the cavity can be expressed as [38]:

(4) $$\frac{dA_{1}}{dt}=\left(j\omega_{1}-1/\tau_{1}-1/\tau_{2}\right)A_{1}+\kappa_{1}S_{1+}+\kappa_{2}A_{2}$$

(5) $$\frac{dA_{2}}{dt}=\left(j\omega_{2}-1/\tau_{2}-1/\tau_{3}\right)A_{2}+\kappa_{3}S_{2+}+\kappa_{2}A_{1}$$

Here *κ*_1_ and κ_3_ are the coupling coefficients between the MIM waveguide and rectangular cavities, and κ_2_ is the coupling coefficient between the left rectangular cavity and right rectangular cavity. Then we can write 1/τ_1_ = κ_1_^2^ and 1/ τ_3_ = κ_3_^2^ as the corresponding decay rates from the cavity to MIM waveguide, and 1/ τ_2_ = κ_2_^2^ is the decay rate from the left cavity to right cavity (or from right cavity to left cavity), ω_1_ and ω_2_ are the resonance frequency of the left and the right resonator, respectively. *j* is the imaginary unit (*j*^2^ = −1). According to energy conservation. The amplitude of the input and the output waves in coupled waveguide should satisfy the following relationships:

(6) $$S_{2-}=j\sqrt{\kappa_{3}}A_{2}$$

(7) $$S_{1-}=S_{1+}+j\sqrt{\kappa_{1}}A_{1}$$

Therefore, the transmittance of the system can be solved from Equations (4)–(7) and can be expressed as:

(8) $$T={\left|\frac{S_{2-}}{S_{1+}}\right|}^{2}={\left|\frac{\kappa_{1}\kappa_{2}\kappa_{3}}{\left[j\left(\omega -\omega_{1}\right)+1/\tau_{1}+1/\tau_{2}\right]\left[j\left(\omega -\omega_{2}\right)+1/\tau_{2}+1/\tau_{3}\right]-\kappa_{2}^{2}}\right|}^{2}$$

When κ_2_ = 0, T = 0. But κ_2_ ≠ 0, the coupling between double rectangular cavities significantly perturbs the wave transmitted from the left waveguide to right waveguide, which result in a complex interference phenomena, and makes the system exhibit a Fano line shape.

## 3. Results and Discussion

**Figure 2:**
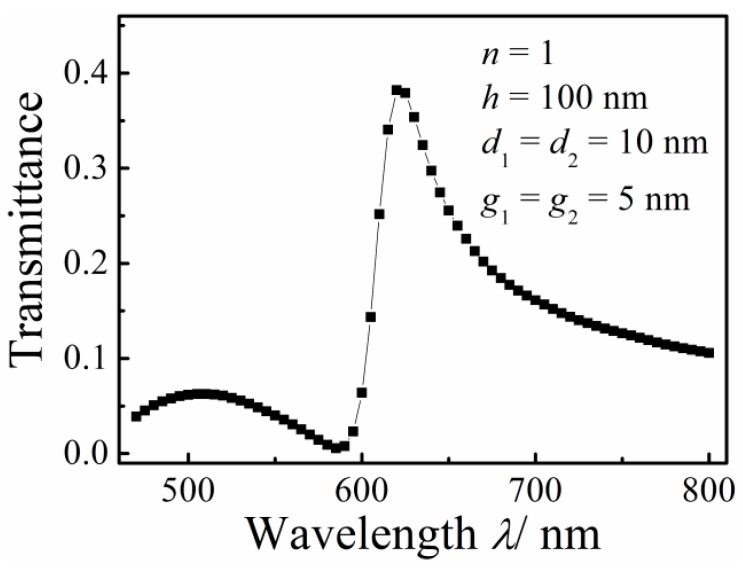
Transmission spectrum of the MIM waveguide-coupled double rectangular cavities.

Figure 2 shows the transmission spectrum of the MIM waveguide-coupled double rectangular cavities with *d*_1_ = *d*_2_ = 10 nm, *h* = 100 nm, *g*_1_ = *g*_2_ = 5 nm and *n* = 1. A resonance peak with asymmetric line shape is observed. The slope of the left shoulder of the resonance peak is obviously larger than that of the right shoulder, which is a typical Fano profile with one maximum and one minimum. The transmittance is near to 0 at the transmission dip (λ = 580 nm). The dip is regarded as a superradiative mode but the peak as a nonoradiative mode.

**Figure 3:**
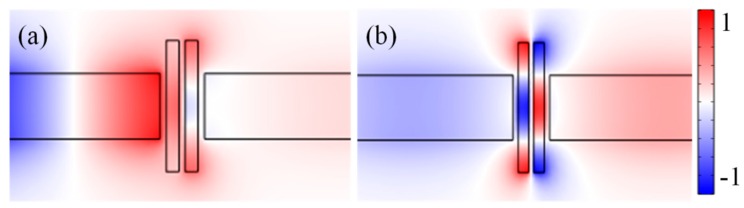
Steady state magnetic field *H*_z_ distributions for the (**a**) transmission dip (580 nm); and (**b**) transmission peak (620 nm) of the MIM waveguide-coupled double rectangular cavities.

Figure 3a,b display the normalized magnetic field *H*_z_ distributions for the transmission dip (λ_dip_ = 580 nm) and transmission peak (λ_peak_ = 620 nm) of the MIM waveguide-coupled double rectangular cavities, respectively. For the superradiative mode (λ_dip_ = 580 nm), the *H*_z_ field distributions show that there is in-phase between the double rectangular cavities. A very weaken coupling occur between double rectangular cavities, and have no SPPs coupled into the right MIM waveguide, which agrees well with the result (κ_2_ = 0, *T* = 0) from Equation (8). For the nonradiative mode (λ_peak_ = 620 nm), the *H*_z_ fields distribution show that there is anti-phase between double rectangular cavities. The superradiative mode is excited by the SPPs from input waveguide, the nonradiative mode cannot. However, it can be excited by the near-field associated with the superradiative mode. Fano resonances arise from the interaction between the superradiative (discrete state) mode and the nonradiative mode (continuum state).

**Figure 4:**
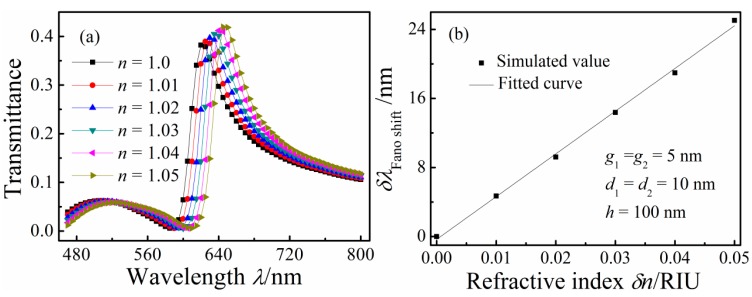
(**a**) Transmission spectra for different refractive index *n*; and (**b**) the shift of the Fano resonance peak as a function of the refractive index change δ*n*.

To investigate the sensitivity of the Fano resonance of the proposed nanosensor to *n*, dielectrics with different *n* were filled in the MIM waveguides and double rectangular cavities. Figure 4a depicts the transmission spectra of the MIM waveguide-coupled double rectangular cavities with *n* of 1–1.05 RIU in increments of 0.01 RIU; others structural parameters *g*_1_, *d*, *h*, and *g*_2_ were fixed at 5, 10, 100, and 5 nm, respectively. With increasing *n*, Re(*n_eff_*) increased. The Fano resonance peak showed a red shift as *n* increased. These Fano resonance peak red shifts can also be explained using Equation (2). The transmission spectra reveal that the transmittance of the Fano resonance peak increased with increasing *n*. Figure 4b shows the shift of the Fano resonance peak as a function of δ*n*. The solid curves are the linear fittings. The sensitivity δλ/δ*n* of the refractive index sensor is 495 nm/RIU according to Figure 4b, and its FOM is 7.5.

**Figure 5:**
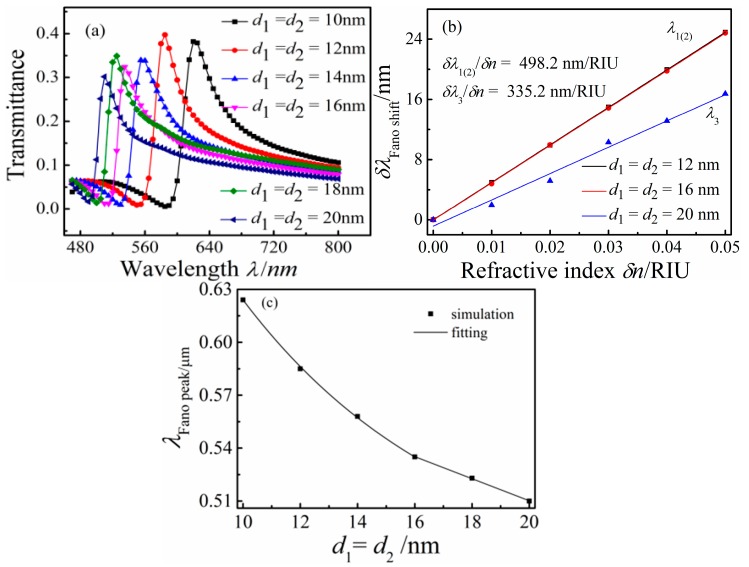
(**a**) Transmission spectra for different rectangular cavity widths *d*_1_(*d*_2_); (**b**) the shift of the Fano resonance peak as a function of the refractive index change δ*n*; and (**c**) the Fano resonance peak as a function of the rectangular cavity widths change *d*_1_(*d*_2_).

To study the effect of the double rectangular cavities widths on the Fano resonance of the MIM waveguide-coupled double rectangular cavities, *d*_1_ and *d*_2_ were increased synchronously from *d*_1_ = *d*_2_ = 10 nm to *d*_1_ = *d*_2_ = 20 nm with fixed *h* = 100 nm, *g* = *g*_1_ = 5 nm, and *n* = 1 RIU. The transmission spectra of the MIM waveguides coupled different double rectangular cavities widths for *d*_1_ = *d*_2_ = 10, 12, 14, 16, 18, and 20 nm are presented in Figure 5a. With increasing double rectangular cavities widths (*d*_1_ = *d*_2_), the Fano resonance of the system blue shifts obviously. This can be explained by the increase of *d*_1_ and *d*_2_ decreasing Re(*n*_eff_), and the blue shift of the Fano resonance peak is also predicted by above Equation (2). Figure 5b shows the shift of the Fano resonance peak (*d*_1_ = *d*_2_ = 12, 16, and 20 nm) as a function δ*n*. The solid curves are the linear fittings. The sensitivity δλ/δ*n* of the nanosensor with *d*_1_ = *d*_2_ = 12 nm is equal to that of the configuration with *d*_1_ = *d*_2_ = 16 nm, with a value of 498.2 nm/RIU. Compared with the nanosensor with *d*_1_ = *d*_2_ = 12 and 16 nm, δλ/δ*n* for the configuration with *d*_1_ = *d*_2_ = 20 nm decreases obviously to 335.2 nm/RIU. As shown in Figure 5c, the Fano resonance peak change as the widths (*d*_1_ = *d*_2_) is an exponential decay function from the *d*_1_ = *d*_2_ = 10 nm to *d*_1_ = *d*_2_ = 16 nm, but it is linear function from *d*_1_ = *d*_2_ =16 nm to *d*_1_ = *d*_2_ = 20 nm. The point (*d*_1_ = *d*_2_ = 16 nm) is a inflection point of the function of Fano peak as the *d*_1_ (*d*_2_), so an abrupt change is observed from *d*_1_ = *d*_2_ = 16 nm to *d*_1_ = *d*_2_ = 20 nm.

**Figure 6:**
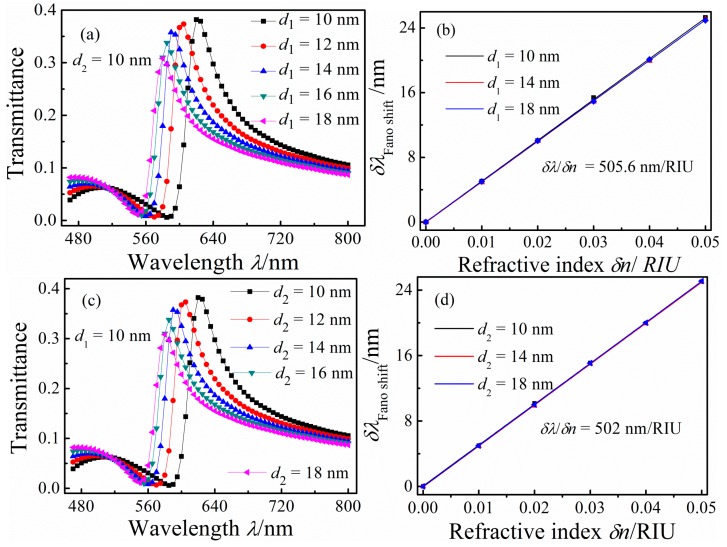
(**a**,**c**) Transmission spectra for different rectangular cavities width *d*_1_ (**a**) and *d*_2_ (**c**); respectively; (**b**,**d**) the shift of the Fano resonance peak as a function of δ*n* with different rectangular cavities width *d*_1_ (**b**) and *d*_2_ (**d**), respectively.

Figure 6a shows that the transmission spectra when *d*_1_ is increased from 10 to 20 nm in 2 nm increments with fixed *d*_2_ = 10 nm, *h* = 100 nm, *g* = *g*_1_ = 5 nm, and *n* = 1 RIU. With increasing *d*_1_, the blue shift and the transmittance of the Fano resonance peak decreased slightly. The increase of *d*_1_ caused Re(*n_eff_*) to decrease in the rectangular cavity. Figure 6b shows the shift of the Fano resonance peak δλ_Fano shift_ as a function of δ*n*, from which we calculated that δλ/δ*n* is 505.6 nm/RIU for this configuration. In addition, we fixed *d*_1_ to investigate the effect of varying *d*_2_ on the transmission spectra. Figure 6c displays the transmission spectra for systems with *d*_2_ = 10, 12, 14, 16, 18, and 20 nm with fixed *d*_1_ = 10 nm. With increasing *d*_2_, the Fano resonance peak blue shifts and its transmittance decreases slightly. Figure 6d presents δλ_Fano shift_ plotted as a function of δ*n* for the configurations with different *d*_2_; δλ/δ*n* determined from this plot is 502 nm/RIU.

**Figure 7:**
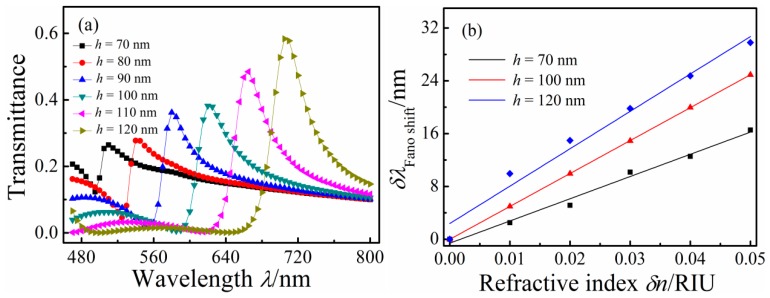
(**a**) Transmission spectra; and (**b**) Fano resonance wavelengths of MIM waveguides-coupled double rectangular cavities with different double rectangular cavities heights *h*.

The simulation indicate that the Fano resonance peak as the changing of the refractive index is linear function, and they parallel to each other nearly for different *d*_1_ or *d*_2_, namely they have same slope. So the refractive index sensitivity δλ/δ*n* curves overlapped each other. To investigate how *h* affects the Fano resonance of the MIM waveguide-coupled double rectangular cavities, transmission spectra for different height *h* of 80, 90, 100, 110, and 120 nm with *g* = *g*_1_ = 5 nm, *d* = 10 nm, and *n* = 1 RIU were calculated, and are plotted in Figure 7a. With increasing *h*, the Fano resonance peak red-shifted linearly because the SPPs pathway in the rectangular cavity lengthened, and its transmittance increased. Figure 7b illustrates the shift of the Fano resonance peak as a function of δ*n* for *h* = 70, 100, 120 nm. The solid curve is the linear fitting. With increasing *h*, δλ/δ*n* increased. For *h* = 70, 100, and 120 nm, δλ/δ*n* are 331, 498, and 596 nm/RIU.

## 4. Conclusions

The transmission properties of MIM waveguide-coupled double rectangular cavities were studied using the FEM. A Fano resonance peak was observed in transmission spectra, and it depended on the mode coupling between the double rectangular cavities. Upon increasing the width of the double rectangular cavities simultaneously, the Fano resonance peak blue shifted and its transmittance decreased, and the sensitivity decreased. Upon increasing the width of one rectangular cavity, the Fano resonance peak blue shifted and the sensitivity did not change. The Fano resonance peak exhibited an obvious red shift and its transmittance increased with increasing height of the double rectangular cavities; the sensitivity also increased obviously. The refractive index sensitivity (596 nm/RIU) of the MIM waveguide coupled double rectangular cavities is higher than the previously [27]. The sensitivity of the proposed sensor based on waveguide coupled resonance is smaller than that of the conventional SPR sensor based on Kretschmann geometry [39], but the former is easier to integrate with various photonic devices and chips than the latter.

## Abbreviations

The following abbreviations are used in this manuscript:

SPPs	Surface plsmon polartions
MIM	Metal-insulator-metal
FEM	Finite element method
FOM	Figure of merit
PML	Perfectly matched layer
3D	Three-dimensional
2D	Two-dimensional
CMT	Coupled mode theory
TM_0_	Fundamental transverse magnetic
FWHM	Full width at half-maximum
